# The Fulham Infirmary

**Published:** 1913-04-05

**Authors:** 


### REPORTS ON POOR-LAW INFIRMARIES.

### THE FULHAM INFIRMARY.

The Fulham Infirmary was established in 1884. In considering these Reports it is essential to bear in mind what is known as Gathorne-Hardy's Act for the Establishment of Asylums for the Sick, Insane, etc., passed on March 29, 1867. That Act, which we hope to deal with on another occasion, created a revolution in the accommodation for the sick poor under the Poor-Law in the Metropolis, and has been attended by momentous changes for the better in many directions. It follows that the year in which each Poor-Law infirmary in the Metropolis was established is important as showing the relative public spirit and capacity of the various Boards of Guardians throughout London forty years ago. Fulham, with the exception of Paddington, was the last of the first fourteen infirmaries erected under Gathorne-Hardy's Act. Structurally, therefore, its buildings should be in advance of the earlier ones, the first of them being Wandsworth and the Poplar and Stepney Sick Asylum. It would be probably unjust and a little hard to criticise the work of the earlier Poor-Law infirmary architects—unjust because in Poor-Law matters the architect is not by any means entirely a free agent, and there seems a wonderful hankering after repetition in types of buildings; a little hard, because some voluntary hospitals, of which the Royal Victoria Infirmary, Newcastle, is a notable instance, though only rebuilt in 1905-6, display the same fault of absence of originality in construction and too close a reproduction from existing types.

At Fulham Infirmary the buildings stand north and south, the northern aspect being that occupied by the offices and quarters of the resident staff, but not of the nurses who are housed in the new home, where they have each a separate room of ample proportions. The nurses' rooms are well furnished, with the exception of the fittings to the furniture supplied by Messrs. Harrod's, and of the design of the tops of the wardrobes which can only have been planned—if mind entered into the transaction—as special receptacles for readily encouraging dust. We certainly think the attention of the directors of Harrod's Stores should be called to this furniture, that they may give instructions to remove the defects it exhibits in the matter of design and fittings. There are now in the market excellent patterns of furniture for hospitals, especially wardrobes, where the tops are aslant and the lodgment of dust is rendered as difficult as possible. We would further call the attention of the Guardians to the wicker chairs in the nurses' sitting-rooms, which might be replaced with advantage by more comfortable and serviceable articles of furniture. We would also suggest that the Guardians might very properly and economically substitute a better quality of china for that at present supplied for the use of the nurses. With these exceptions the new home is satisfactory, being well planned, airy, and having the great merit of a separate kitchen with a most attractive refectory, well proportioned, well lighted, and suitably decorated. A separate kitchen is essential to the economical administration of the nurses' home in any large hospital, *providing the matron has the power vested in her to reject all supplies which are either bad or unsatisfactory when delivered.* We consider that the plan and arrangements of this nursing home are a credit to the architect and to the Guardians.

The general plan of this infirmary is a straight corridor with many of the wards to the south of it and the day-rooms, administration, and other buildings to the north. The large wards contain from 32 to 36 beds, and the average number of patients to each nurse, calculating the total patients and the whole day and night staff, is about seven patients to one nurse. The beds in the wards are coupled, the wall space is inadequate in the hospital sense, and the floor space too, as the wards are, we learn, only 22 feet in width, which though adequate in a small ward of from 8 to 10 beds is at least 4 and probably, as we hold, 6 feet too narrow for great wards like those at Fulham. At the time of our visit the windows were open top and bottom in most of the wards, which were remarkably clean and well kept, and the atmosphere was satisfactory.

The fittings, on the whole, were in good order, though the number of basins provided for washing purposes seemed to us too few for the size of the wards. We observed, too, that in the baths in the new nurses' home, owing to the inlet for the water being placed at the top instead of at the bottom of the bath, the enamel had already worn away, making the baths look unsightly in consequence. This is a matter which the manufacturers should put right by changing the fittings, if their attention be called to it at the proper time.

We observed that, although this infirmary was planned with day-rooms, at the present time there are no day-rooms in fact, the whole of them having apparently been changed into wards, as the pressure on beds has increased. The arrangements in the maternity department were attractive and comfortable, and the regulations and care taken to isolate, where necessary, and to protect this class of patients from undesirable influences and associates, are good. One noticeable feature which increases our desire to see these great infirmaries divorced altogether from any pauper complexion is the fact that the number of married women entering the infirmary for childbirth shows a remarkable and steady increase.

The operation theatre is so planned as to make this unit as simple and easily worked as possible. Several of the devices introduced by the superintendent, Dr. Thackray Parsons, have considerable practical value. Everything in this department was in good order and smartly kept. It is creditable to the resident medical staff and to the Guardians that this infirmary should have a department for massage, electricity, and medical gymnastics, in charge of a special sister who devotes the whole of her time to this work, and is assisted by two probationers during the busy portions of the day. It contains a Dowsing radiant-heat bath, which has produced excellent results by lessening the suffering and increasing the comfort of several cases which have been bedridden for years. Frenkel's exercises are here systematically used, and the department has justified its existence by proving that several patients have been able to take their discharge who, without its help, must have remained permanent inmates of the infirmary or workhouse. Another good result from it has been the reduction of time and treatment required in cases of fractures, sprains, paralytic and arthritic conditions. Dr. Parsons, with the co-operation of the Guardians, is steadily improving the facilities for treatment and diagnosis, one of the latest additions being a well-equipped and ample chamber for Röntgen rays and other diagnostic purposes.

It was satisfactory to find that after thirty years' wear and tear this infirmary is structurally well maintained. We noted one novelty in the lavatories of the nursing home—namely, the supply of a separate stopcock for each supply pipe to every basin. Does Mr. Saxon Snell find this plan from its utility to justify the extra cost? The wish of the Guardians to encourage efficiency was demonstrated by the recent addition of some excellently-planned linen rooms in connection with each ward unit. The matron may be warmly congratulated upon obtaining these rooms. We noted, however, that the stock of linen is evidently kept down to the lowest possible point. We found the dispensary excellently kept, and so arranged as to facilitate the work of the dispensers as much as possible. We did not inspect all the wards, but those which we inspected were plastered and attractively painted; the floors were in surprisingly good condition, except in one case where maple veneer had been placed over the old boards, when, owing to the quality of the maple, the results were not so good as they might and ought to be.

Taking this infirmary and looking at it as a whole and in detail we are of opinion that by reducing the number of beds in the larger wards, by adding new lockers, which are badly needed in substitution for the rather objectionable type of the old locker at present in use, and by the introduction of a few modifications, the cost of which would not be serious, its buildings might be easily brought up to a level which would enable the infirmary to be used satisfactorily and justly as a public hospital. The condition of the bedsteads, the bedding, and the linen throughout the infirmary was good, though we think the hair bolsters or many of them should be sent away to be remade. This practice is adopted already, but the numbers sent seem to us to be too few to enable the whole of the bedding to be kept in that high state of relative comfort and efficiency which characterises the best-administered hospitals in the present day. Our congratulations are due to the whole of the management and to everyone who has had a hand in maintaining the present high level of efficiency which this institution exhibits after thirty years' continuous working. Miss J. F. Ballantyne is to be congratulated upon the type of probationers whom she has succeeded in securing, and we have little doubt that the excellence of the nursing accommodation and the thoroughness of Miss Ballantyne's system are calculated to maintain this high standard at this infirmary.

